# Microbiological Parameters in the Primary Production of Berries: A Pilot Study

**DOI:** 10.3390/foods7070105

**Published:** 2018-07-05

**Authors:** Guerrino Macori, Giovanna Gilardi, Alberto Bellio, Daniela Manila Bianchi, Silvia Gallina, Nicoletta Vitale, Maria Lodovica Gullino, Lucia Decastelli

**Affiliations:** 1 Food Control and Production Hygiene Unit, Istituto Zooprofilattico Sperimentale del Piemonte, Liguria e Valle d’Aosta, via Bologna 148, 10154 Turin, Italy; alberto.bellio@izsto.it (A.B.); manila.bianchi@izsto.it (D.M.B.); silvia.gallina@izsto.it (S.G.); lucia.decastelli@izsto.it (L.D.); 2 Centre of Competence for Innovation in Agro-Environmental Field, Agroinnova, University of Torino, Largo Paolo Braccini 2, 10095 Grugliasco (TO), Italy; giovanna.gilardi@unito.it (G.G.); marialodovica.gullino@unito.it (M.L.G.); 3 Istituto Zooprofilattico Sperimentale del Piemonte, Liguria e Valle d’Aosta, via Bologna 148, 10154 Turin, Italy; nicoletta.vitale@gmail.com

**Keywords:** fresh berries, Hepatitis A virus, virological quality, molecular detection

## Abstract

The primary production of fresh soft fruits was considered to be a suspected critical point for the contamination of frozen berries that were responsible for the large 2013–2014 Hepatitis A virus (HAV) outbreak in Europe. In this study, an Italian berries’ production area was studied for its agro-technical characteristics, and the fresh fruits were analyzed for the presence of enteric viruses (HAV and Norovirus (NoV) genogroup I and genogroup II (GGI and GGII)), the enumeration of hygienic quality parameters, and the prevalence of bacterial pathogens. A total of 50 producers were sampled, who specialized in the exclusive or shared cultivation of berries. *Escherichia coli* was detected in two blackberry samples, whereas HAV and Norovirus were not detected. The samples were negative for *Salmonella* spp., *Listeria monocytogenes*, and Shiga toxin-producing *Escherichia coli* (STEC). The farms’ attributes were not associated with positive samples, apart from the presence of *E. coli* and the aerobic mesophilic bacteria for blackberry that were statistically correlated. In blueberries, the high aerobic mesophilic count could likely be associated with the resistance of the outer layer to handling. However, the two pathogens (*Salmonella* spp. and STEC) and the targeted viruses (HAV, NoV GGI and GGII) were not detected, highlighting the low risk of foodborne pathogens and viral contamination at the primary production stage of the berry food chain in the area considered in this pilot study.

## 1. Introduction

Berries, such as raspberries (*Rubus idaeus*), blueberries (*Vaccinium myrtillus*), blackberries (*Rubus fruticosus*), redcurrant (*Ribes rubrum*), and lingonberries (*Vaccinium vitis-idaea*), represent an important type of fresh produce in Europe, in terms of production volume and economic value; however, little information is available about their accompanying microbiological risks [1,2]. The fruits are often consumed after minimal processing, and the production consists of various approaches and culturing techniques, including cultivation in soil, in pots, by hydroponic technologies, in open fields, and in greenhouses. Berries can be produced in exclusive or shared cultivations, and the size of the farms can vary from large- to small-scale, based on the production requirement. In addition, the production may include private gardens, and fruits can be harvested in the wild [3]. Italy is one of the main producers of berries in Europe. In 2014, there were 135,320 tonnes of strawberries produced; 2465 tonnes of raspberries; 1667 tonnes of blueberries; 783 tonnes of currants; and 102,313 tonnes of “berries nes”, which include blackberries (*Morus nigra*), loganberries, white and red mulberries (*Myrtus alba*; *M. rubra*), myrtle berries (*M. communis*), huckleberries, and dangleberries (*Gaylussacia* spp.) [4]. Outbreaks of foodborne illness due to the consumption of fresh produce contaminated with human pathogens are increasing, probably linked to the fact that these products are usually consumed raw [5,6,7,8]. Berries were responsible for foodborne outbreaks due to virus contamination, including as frozen fruits [9,10,11]; frozen raspberries have been reported in previous studies as contaminated produce responsible for Norovirus outbreaks in Europe [12,13,14]. Other important outbreaks highlighted that frozen berries are an efficient vehicle of enteric virus infection, as in the case of the multi-country outbreaks of Hepatitis A Virus (HAV) reported in 2013 [15]. These cases were important challenges for the surveillance systems of microbial infections [16], and for the establishment of interdisciplinary task forces for the management of emergencies [17].

Numerous risk factors are considered to be responsible for the contamination of berries, such as the type of cultural techniques (i.e., traditional soil or hydroponic cultivations) and the type of irrigation (i.e., lowered well-water or flooded surface channel water). In addition, the harvesting of small fruits mostly takes place by hand, a demonstrated vehicle by which human pathogenic viruses enter the berry fruit chain [18,19]. Among fresh produce, strawberries were also reported as a novel vector for Shiga toxin-producing *Escherichia coli* (STEC) infection, including serogroup O157:H7, due to wildlife fecal contamination [20,21]. 

Animal reservoirs can also be responsible for the spread of STEC O157:H7 [22], where the soil can be a pathway for the contamination of irrigation water [23,24]. In a study conducted in Belgium that involved six strawberry producers, the authors included irrigation water samples that tested positive for three of the top five non-O157 serogroups (O26, O145, O103) [25], suggesting that other pathogenic strains of *E. coli* can be associated with fresh strawberries. Moreover, contamination can also occur at the farm level, where animals are considered the primary source of contamination [8,11,18,26,27], especially where mixed crop–livestock farms (MCLFs) are carried out [28]. This type of farming is practiced on small farms, where livestock can be in contact with the grown crops, or in organic farms, where the composted animal waste can be used to fertilize the soil for growing crops [29]. 

The evaluation of risk factors directly at the primary production is considered a central key point, because fresh fruits can be introduced in the market as ready-to-eat food. This approach has been applied to other foods of non-animal origin, which are minimally processed and whose production may not include processing steps or control points that will ensure inactivation or removal of microbial and viral contaminations [30]. The identification of critical points at the primary production can be achieved with the use of surveys, according to audit questions of regulatory agencies for the Good Agricultural Practices (GAP) requisitions [31], in order to identify the risk factors in the fresh produce sector.

The studies at the primary production level have been poorly documented, despite the high risk factors related to enteric viruses and pathogens responsible for food-borne outbreaks in berries, and the prevalence of pathogens in berries in relation to the field characteristics is not currently available [28]. More availability of these data would greatly improve the knowledge on the hygienic conditions in this production sector. 

In this pilot study, we evaluated freshly harvested berries for the presence of the HAV, Norovirus (NoV) genogroup I and genogroup II (GGI and GGII); human enteric bacteria, including *E. coli* and STEC O157:H7; *Listeria monocytogenes*; *Salmonella* spp.; and other microbial parameters, such as the enterococci and the aerobic mesophilic bacteria. The risk related to the primary production sites was evaluated by collecting information at the farm level, through a specific on-site questionnaire. The survey included growing conditions, irrigation of the crops, water distribution systems, and a specific question related to the farm characteristics and workers that can be considered for the appropriate implementation of food safety management systems in the farms.

## 2. Materials and Methods 

### 2.1. Collection of Data and Sampling Strategy

The sampling plan was developed using background information questionnaires, and confirmed through the direct observation of conditions and practices at farm level. The data collected included the characteristics of the growing system and the production features: berry species, farm sizes, cultural conditions (soil/pots, unprotected in open fields or protected in plastic tunnels), type of water used, and irrigation conditions. In addition, there were specific questions regarding farm management (i.e., training of the workers) and level of practice for a mixed crop–livestock farm (MCLF) production system (i.e., separated farming, rotational farming, and fully combined farming). The farmers were asked specific questions in the case of organic farming, including certification status, the agency of certification, and fertilization practices: the type(s) of manure or compost or chemicals, the age of manure or compost, and the time of application. The questionnaire requested information on handling practices during harvesting, as well as subsequent handling operations, like washing, packaging, and storage. The information collected from the survey was used to identify associations with the microbiological results. 

The farms were sorted by zip code and selected to provide broad geographical representation of producers throughout the production area. Eighty-three berry producers were willing to participate this study, and the results of the questionnaires were taken into account in order to choose the best statistical approach to sample the selected farms. 

The berry samples consisted of 400 g of whole fruit sampled wearing gloves and placed in sterile bags (RollBag, Interscience, St. Nom., France). All the samples were transferred to the laboratory in a controlled temperature box, stored at 4 °C, and analyzed within 24 h. The berry samples were transferred in sterile blender bags (BagFilter, Interscience) and immediately processed. Samples with individual fruits larger than 2.5 cm × 2.5 cm × 2.5 cm were coarsely cut and placed into the sterile blender bags in smaller pieces using a sterile blade.

### 2.2. Microbiological Analysis and RNA Extraction

The berry samples were analyzed to establish the presence or absence of *Salmonella* spp., *L. monocytogenes*, and Shiga toxin-producing *E. coli* (STEC). Enumeration of *E. coli*, aerobic mesophilic count (AMC), and *Enterobacteriaceae* were used as hygiene indicator parameters. The analyses were performed according to methods and standard procedures shown in Table 1. 

RNA was extracted following the ISO/TS 15216-2 method for NoV and HAV detection in berries (soft fruits) and vegetables [41]. The pellet suspension obtained from the washing protocol was pre-treated by the chloroform–butanol method, and the viral genome RNA was extracted and purified with QIAamp UltraSens Virus Kit (Qiagen, Milan, Italy), according to the manufacturer’s recommendation; the samples obtained were stored at −20 °C.

### 2.3. Two Step Reverse Transcription Seminested-PCR (RT-PCR) and Quantitative Real-Time Reverse Transcription-PCR (Real-Time RT-PCR)

The RNA from samples was extracted for the following target viruses: HAV, NoV GGI and NoV GGII, following the one-step real-time RT-PCR method described in the ISO/TS 15216-2. The process control virus was detected with specific primers and probes [43]. HAV was also detected using conventional reverse transcription-PCR (RT-PCR) followed by a seminested-PCR assay, according to the previously described procedure [42]. Process control virus for the extraction and positive and negative controls for PCR were included.

### 2.4. Statistical Analysis

The experiments were carried out according to the sampling plan. Bacteria counts (AMC, *Enterobacteriaceae* and *E. coli*) were converted to log colony forming unit (CFU), and were expressed as mean ± standard deviation (SD). To evaluate the relationship between the three bacteria, the Spearman’s rank correlation rho was calculated as a measure of correlation. The prevalence of AMC, *Enterobacteriaceae*, and *E. coli* was calculated as well at 95% confidence interval (CI)) by binomial exact methods. The chi square test (χ^2^) was applied to test the associations between the presence of bacteria and the following farm features: growing systems, berry species, farm size, cultural conditions (soil/hydroponic cultivation, unprotected in open fields or protected in plastic tunnels), and the type of water used for irrigation. Categorical variables were reduced to as few categories as possible, ideally two. The univariate odds ratios were calculated. A multivariate logistic regression model was used to estimate the association between study factors to control for confounding. Statistical modeling was initially performed by bivariate analysis in order to select relevant factors. The significance of factors was tested using Pearson’s χ^2^ test. Only factors presenting *p*-value < 0.10 were considered in multivariate models. All analyses were performed using R Studio (RStudio, Inc., Boston, MA, USA).

## 3. Results

### 3.1. Collection of Data and Sampling Strategy

**Figure 1:**
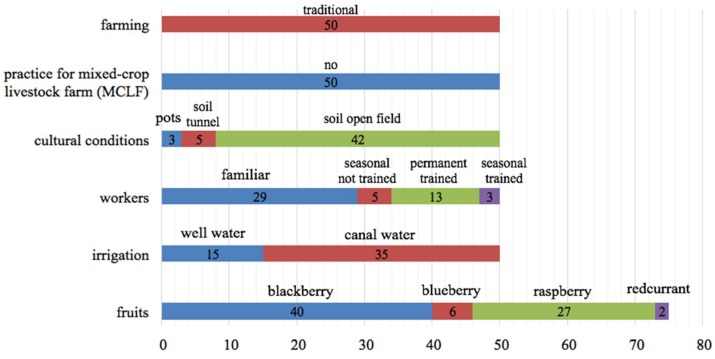
Results of the questionnaire.

Based on the complete information collected at the farm level, 50 producers from the 83 survey participants were selected for the sampling phase, and were organized according to farm sizes. The small producers provided one fruit sample, and the large producers provided two fruit samples, giving a total of 75 berry batches. This strategy allowed the collection of a single sample from 25 producers with farms from 300 to 1999 m^2^ in size, and two samples from producers with large farms (greater than 2000 m^2^) at different harvest times. The results collected by questionnaires are presented in Figure 1.

### 3.2. Microbiological Measurements and Risks Related to Crop Management 

The data representing the features of the farms and the results of the microbiology analysis are presented in Table 2. All the samples tested negative for the RNA of the targeted viruses: HAV, NoV GGI, and NoV GGII. *Salmonella* spp., *L. monocytogenes*, and STEC resulted negative. The AMC ranged from between 1.7 and 6.9 log CFU/g (50 < AMC < 8.1 × 10^6^) for 61 samples. 

**Figure 2:**
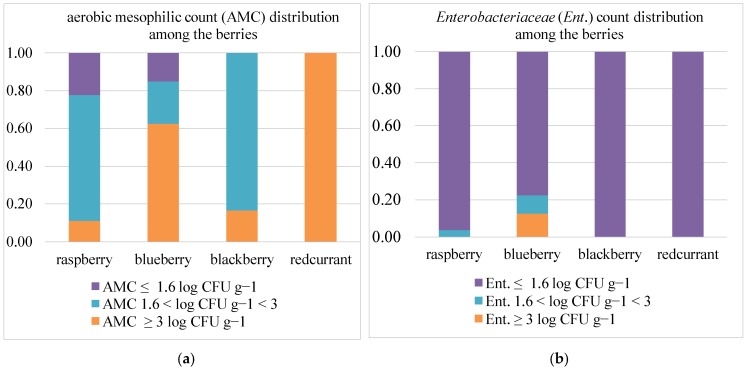
Microbiological analysis and distribution among berries: (**a**) aerobic mesophilic count (AMC); (**b**) *Enterobacteriaceae* (*Ent*.).

Two blueberry samples, as well as one blackberry and one raspberry sample, ranged between 1 and 1.6 log CFU/g, while six raspberry and four blueberry samples resulted as <1 log CFU/g. The *Enterobacteriaceae (Ent.)* counts ranged from between 1 log and 4 log CFU/g in ten samples. Seven blueberry samples ranged between 1 and 1.6 log CFU/g, and 58 samples resulted as <1 log CFU/g, including raspberries (26 samples), blueberries (24 samples), blackberries (6 samples) and redcurrants (2 samples). *E. coli* was only found in two blackberry samples with a value of 1.8 log CFU/g and 4.6 log CFU/g, respectively. The AMC and *Ent.* results were combined according to three value levels: (i) the highest level (value ≥ 3 log CFU/g); (ii) the central value (1.6 < value < 3 log CFU/g); and (iii) the low level (value < 1.6 log CFU/g) (Figure 2a,b). 

*Enterobacteriaceae* and *E. coli* were detected in blackberries and raspberries at a concentration higher than 3.0 log CFU g^−1^. 

Log CFU descriptive statistics for AMC, *E. coli* and *Ent.* are shown on Table 3. The Spearman’s rank correlation rho showed a moderate correlation between AMC and *E. coli* (rho = 0.55, *p*-value < 0.0001), while a weak correlation was found between *E. coli* and *Ent*. (rho = 0.37, *p*-value = 0.001) and between AMC and *Ent*. (rho = 0.27, *p*-value = 0.02). The prevalence of *Ent.*-positive berries was estimated to be 22.7% (17/75), while for *E. coli* the prevalence was 2.7% (2/75) and for AMC was 86.7% (65/75).

The association between the presence of *Ent*. and berry species was shown to be statistically significant via a chi-square test (Table 4), while others factors were shown to be not significant. This association was also confirmed by logistic regression (χ^2^ = 13.7, degrees of freedom = 2, *p*-value < 0.001); blackberry had nearly 23 times (OR 22.67; CI 95% 4.19–422.93) the probability to be *Ent*.-positive than other berries.

## 4. Discussion

The farms that participated in the survey presented here ranged from between 300 and 1999 m^2^. In order to correctly represent the berry production sectors, the sampling strategy consisted in sampling one batch of fresh produce collected from each small producer, and two batches from each large producer. This allowed for the collection of 75 fruit samples, which overall is a heterogeneous representative sample of the different berries produced in the area considered. The sample results were negative for HAV, *Salmonella* spp., and STEC. Blackberry was the only fruit found positive for *E. coli*, and compared with the other berries, was the most *Enterobacteriaceae*-positive (Table 1). Despite the high AMC and the high value for *Enterobacteriaceae* in the tested fruits, there were no reported visible alterations that could have affected the commercialization, whereas Ragaert and colleagues described a significant correlation between the organoleptic alteration of vegetables and bacterial counts higher than 7 or 8 log CFU/g [44]. However, we highlighted the association between the presence of *E. coli* and the aerobic mesophilic bacteria for blackberries. This result suggests the use of AMC as a predictor parameter for the presence of *E. coli* in this ready-to-eat-fruit for the dataset presented in this study. Among all the berries analyzed, blackberries and raspberries showed the highest AMC and *Enterobacteriaceae*; this could be justified by the different surface structure observed, which may affect the attachment of the bacterial cells [45]. 

The European Food Safety Authority (EFSA) has highlighted the need to provide scientific requirements for the identification of the main risk factors for *Salmonella* spp. and norovirus in berries [30]. In this study, we tested the combination of risks that can influence the occurrence of pathogens in each berry farm environment. The questionnaire used for the collection of the farm characteristics highlighted the differences among the production farms and the evaluation of the risk included the environmental factors, in particular the proximity to animal rearing and the direct contact with animal reservoirs (domestic or wildlife) gaining access to berry fields. According to the EFSA’s opinion, other factors were evaluated for the risk assessment: the use of untreated or insufficiently treated manure or compost and the type of water used for irrigation. The farms that participated in this study were all “traditional”, in fact were not used treated manure or compost and all the farms were not mixed crop–livestock that might have had a risk of fecal contamination of the crops.

The present study was designed to provide an initial estimation on the microbiological quality of fresh berries. Some of the characteristic features were the random collection of samples, the geographical distribution, and the diversity of the farms in terms of water collection and type agricultural technique. However, our results could have been influenced by the unbalanced numbers of samples among produce varieties. The producers that participated in this study practiced traditional farming; in fact, there were no organic producers or the use of manure and compost, and the participants were not mixed crop–livestock farms with a resulting diminished risk of fecal contamination. Additional work is needed to generate a more comprehensive data set that would address the influence of those variables on bacterial populations and the potential for foodborne disease risks. The results of the questionnaire were used for the implementation of food safety management systems, including good agriculture practices (GAP) at farm level, good hygiene practices (GHP), and good manufacturing practices (GMP) at the packaging and delivery company. These practices aim to control and reduce the microbial contamination of foods, and manage the spread or further growth of microorganisms if contamination has occurred [46].

In terms of highest contamination, the combination of different producers and the characteristics of the crops are not supportive of common causative features. In fact, distinct contaminated samples did not share demonstrated mutual attributes. This could perhaps indicate multiple incidences or sources of contamination, highlighting an urgent need to further improve hygiene practices during berry production. As demonstrated for raspberries, new technologies for the decontamination of fresh fruits will increase the safety of these products in the future [47]. As suggested by other authors in a previous study [28], *E. coli* can be used as an indicator of recent human or animal fecal contamination, but microbiology monitoring should be done frequently, and other technological parameters must be considered in the evaluation of the quality. All these factors are important for the good agricultural practices (GAP) and good hygienic practices (GHP), with regards to improvement of a farm. In this study, we included the record of several parameters that were collected by questionnaire in the production and harvesting sites. The type of water and irrigation, the training for the product’s handlers, and the type of cultivation are all parameters that we suggest should be included in the GAP and GHP manuals, for their importance during the evaluation of the risk and traceability in the event of a foodborne outbreak 

## 5. Conclusions

In our pilot study, the high AMC value associated with blueberries could likely be correlated with the higher resistance of the outer layer to handling. These values can be considered a microbial contamination, but there are no guidelines for the limits of these microbiological parameters on fresh produce [37]. However, the two pathogens (*Salmonella* spp. and STEC) and the targeted viruses (HAV, NoV GGI and GGII) were not detected, thus highlighting the low risk of foodborne pathogens and viral contamination at the primary production stage of the berry food chain in the area considered, underpinning the safety of this sector. In conclusion, the information collected with the questionnaire and its combination with the microbiology results helped to draw a consistent picture of the berry-producing sector that could be used in future risk assessment studies. 

## Figures and Tables

**Table 1:** Methods used for the microbiological analysis.

Parameter	Method	Reference
*Salmonella* spp.	Real-time polymerase chain reaction (Real-time PCR)	[32,33]
	Culture technique	[34]
*Listeria monocytogenes*	Real-time PCR	[35]
	Culture technique	[36]
Shiga Toxin-producing *E. coli* (STEC)	Real-time PCR	[37]
	Culture technique	[37]
Aerobic Mesophilic Count	Culture technique	[38]
*Enterobacteriaceae*	Culture technique	[39]
*Escherichia coli*	Culture technique	[40]
hepatitis A virus (HAV) and norovirus genogroup I and II (Nov GGI/GGII)	Real time-PCR	[41,42]

**Table 2:** Characteristics of the farm involved in the sampling plan and results of the microbiology analysis.

Farm Characteristics and Results of the Questionnaires							Results Microbiology Analysis (CFU/g)		
Id	Prod	Fruit	Farm Size	Irrigation	Workers	Cultural Conditions	AMC	*E. coli*	*Ent.*
1	1	blackberry	B	well water	ST	soil open field	14,000	<10	1500
2	1	blackberry	B	well water	ST	soil open field	1500	<10	<10
3	2	blackberry	B	well water	ST	soil open field	130	<10	<10
4	2	blackberry	B	well water	ST	soil open field	1200	<10	<40
5	3	raspberry	B	well water	ST	soil open field	2900	<10	<10
6	3	raspberry	B	well water	ST	soil open field	760	<10	<10
7	4	blackberry	B	canal water	fam	soil open field	9100	<10	<40
8	4	blackberry	B	canal water	fam	soil open field	1000	<10	<10
9	5	blackberry	S	canal water	fam	soil open field	290	<10	<10
10	6	raspberry	S	canal water	fam	soil open field	320	<10	<10
11	7	blueberry	S	canal water	fam	soil open field	580	<10	<10
12	8	red currant	S	canal water	SNT	soil open field	2900	<10	<10
13	9	raspberry	B	canal water	SNT	soil open field	160	<10	<10
14	9	raspberry	B	canal water	SNT	soil open field	250	<10	<10
15	10	raspberry	B	canal water	fam	Pots	310	<10	<10
16	10	raspberry	B	canal water	fam	Pots	<10	<10	<10
17	11	blackberry	B	canal water	SNT	soil tunnel	150	<10	<10
18	11	blackberry	B	canal water	SNT	soil tunnel	<40	<10	<10
19	12	blackberry	S	canal water	fam	soil open field	<10	<10	<10
20	13	blackberry	B	canal water	fam	soil open field	<40	<10	<10
21	13	blackberry	B	canal water	fam	soil open field	2200	<10	<10
22	14	blackberry	S	canal water	fam	soil open field	1500	<10	<40
23	15	blackberry	S	canal water	fam	soil open field	1000	<10	<10
24	16	blueberry	B	canal water	fam	soil open field	98,000	<10	<10
25	16	blueberry	B	canal water	fam	soil open field	<400	<10	<10
26	17	blackberry	B	canal water	PT	soil open field	600	<10	<10
27	17	blackberry	B	canal water	PT	soil open field	35,000	60	1500
28	18	raspberry	S	canal water	fam	soil open field	700	<10	<10
29	19	raspberry	S	canal water	fam	Pots	800	<10	<10
30	20	blackberry	S	well water	fam	soil open field	700	<10	<10
31	21	red currant	S	canal water	fam	soil open field	8800	<10	<10
32	22	blackberry	S	canal water	fam	soil open field	5200	<10	<10
33	23	raspberry	S	canal water	fam	soil open field	50	<10	<10
34	24	blackberry	B	canal water	PT	soil tunnel	260	<10	<10
35	24	blackberry	B	canal water	PT	soil tunnel	18,000	<10	<40
36	25	raspberry	S	canal water	SNT	soil open field	50	<10	<10
37	26	blackberry	B	canal water	fam	soil open field	22,000	<10	<10
38	26	blackberry	B	canal water	fam	soil open field	1400	<10	<40
39	27	blackberry	S	canal water	fam	soil open field	17,000	<10	<10
40	28	blackberry	S	well water	fam	soil open field	1500	<10	<40
41	29	raspberry	S	canal water	fam	soil open field	660	<10	<10
42	30	raspberry	B	canal water	fam	soil tunnel	500	<10	<10
43	30	raspberry	B	canal water	fam	soil tunnel	100	<10	<10
44	31	raspberry	B	canal water	fam	soil open field	280	<10	<10
45	31	raspberry	B	canal water	fam	soil open field	<10	<10	<10
55	32	raspberry	B	well water	fam	soil open field	100	<10	<10
46	32	raspberry	B	canal water	fam	soil open field	<10	<10	<10
47	33	raspberry	S	canal water	fam	soil open field	<10	<10	<10
48	34	blackberry	S	canal water	SNT	soil open field	<10	<10	<10
49	35	raspberry	S	canal water	fam	soil open field	600	<10	<10
50	36	blackberry	S	canal water	fam	soil open field	1000	<10	<10
51	37	raspberry	S	canal water	fam	soil open field	1900	<10	<400
52	38	blackberry	S	canal water	fam	Pots	250	<10	<10
53	39	blackberry	B	well water	PT	soil open field	43,000	<10	9900
54	39	blackberry	B	well water	fam	soil open field	2400	<10	<10
56	40	blackberry	B	canal water	PT	soil open field	9600	<10	<10
57	40	blackberry	B	canal water	PT	soil open field	8,100,000	45,000	50,000
58	41	blackberry	B	canal water	PT	soil open field	1500	<10	<40
59	41	blackberry	B	canal water	PT	soil open field	43,000	<10	150
60	42	blackberry	S	well water	fam	soil tunnel	8600	<10	<40
61	43	raspberry	B	canal water	PT	soil open field	4600	<10	<10
62	43	raspberry	B	canal water	PT	soil open field	900	<10	<10
63	44	blackberry	S	well water	PT	soil open field	120	<10	210
64	45	raspberry	B	well water	PT	soil open field	<10	<10	<10
65	45	raspberry	B	well water	PT	soil open field	<10	<10	<10
66	46	blackberry	B	well water	PT	soil open field	100	<10	<10
67	46	blueberry	B	well water	PT	soil open field	180	<10	<10
68	47	blackberry	B	well water	PT	soil tunnel	<10	<10	<10
69	47	blackberry	B	well water	PT	soil tunnel	20,000	<10	700
70	48	blackberry	B	well water	PT	soil open field	<10	<10	<10
71	48	blackberry	B	well water	PT	soil open field	12,000	<10	3800
72	49	raspberry	B	well water	PT	soil open field	<400	<10	<10
73	49	raspberry	B	well water	PT	soil open field	180	<10	<10
74	50	blueberry	B	well water	PT	soil open field	110	<10	<10
75	50	blueberry	B	well water	PT	soil open field	410	<10	<10

Id: number of identification, prod: producer, B: big farm (size of farm equal or greater than 2000 m^2^), S: small farm (size of farm equal or greater than 300 m^2^ to a size of 1999 m^2^), ST: seasonally trained, SNT: seasonally not trained, fam: familiar, PT: permanent trained, pots: cultivation in pots, AMC: aerobic mesophilic count, *Ent.*: *Enterobacteriaceae.*

**Table 3:** Log CFU descriptive statistics for aerobic mesophilic count, *Escherichia coli*, and *Enterobacteriaceae*. Bacteria counts were converted to log CFU and were expressed as mean ± standard deviation (SD). The prevalence of the parameters was calculated, as well as 95% confidence intervals (CI 95%) by binomial exact methods.

Bacteria	Median	Mean	SD	Range	Max
Log Aerobic Mesophilic Count	2.778	2.801	1.140	5.910	6.908
Log *Enterobacteriaceae*	1.000	1.059	0.430	3.650	4.653
Log *Escherichia coli*	1.000	1.325	0.760	3.700	4.699

**Table 4:** Association between the presence of *Enterobacteriaceae (Ent.)* and farm characteristics estimated by a chi-square test. A *p*-value < 0.05 is considered to be statistically significant.

Factor	Level	*N*	*Ent.*	%	*χ* ^2^	Df	*p*-Value
Berry	Blackberry	56	16	28.6%	12.649	1	0.000
	Others	19	1	5.3%			
Workers	Trained	22	11	50.0%	3.278	1	0.070
	Not Trained	36	6	16.7%			
Farm Size	Small	25	5	20.0%	0.010	1	0.922
	Big	50	12	24.0%			
Water Type and Irrigation	Canal water	50	9	18.0%	1.150	1	0.283
	Well water	25	8	32.0%			
Cultural Conditions	Pots	4	0	0.0%	1.757	2	0.415
	Soil Open Field	62	14	22.6%			
	Soil Tunnel	9	3	33.3%			

*N*: value of the parameter, Df: degrees of freedom.
